# Delayed local reaction at a previous injection site reaction with dupilumab

**DOI:** 10.1002/rcr2.852

**Published:** 2021-09-21

**Authors:** Toshiyuki Sumi, Yuta Nagahisa, Keigo Matsuura, Motoki Sekikawa, Yuichi Yamada, Hisashi Nakata, Hirofumi Chiba

**Affiliations:** ^1^ Department of Pulmonary Medicine Hakodate Goryoukaku Hospital Hakodate Japan; ^2^ Department of Respiratory Medicine and Allergology Sapporo Medical University School of Medicine Sapporo Japan

**Keywords:** bronchial asthma, dupilumab, injection site reaction, polysorbate, severe asthma

## Abstract

Clinicians should be aware that biologic agents, which include polysorbates, can cause delayed local skin reactions at a previous injection site.

## Graphical abstract

This case highlights that biologic agents, which include polysorbates, can cause delayed local skin reactions at a previous injection site.

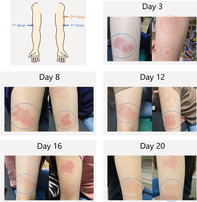

## CLINICAL IMAGE

**FIGURE 1:**
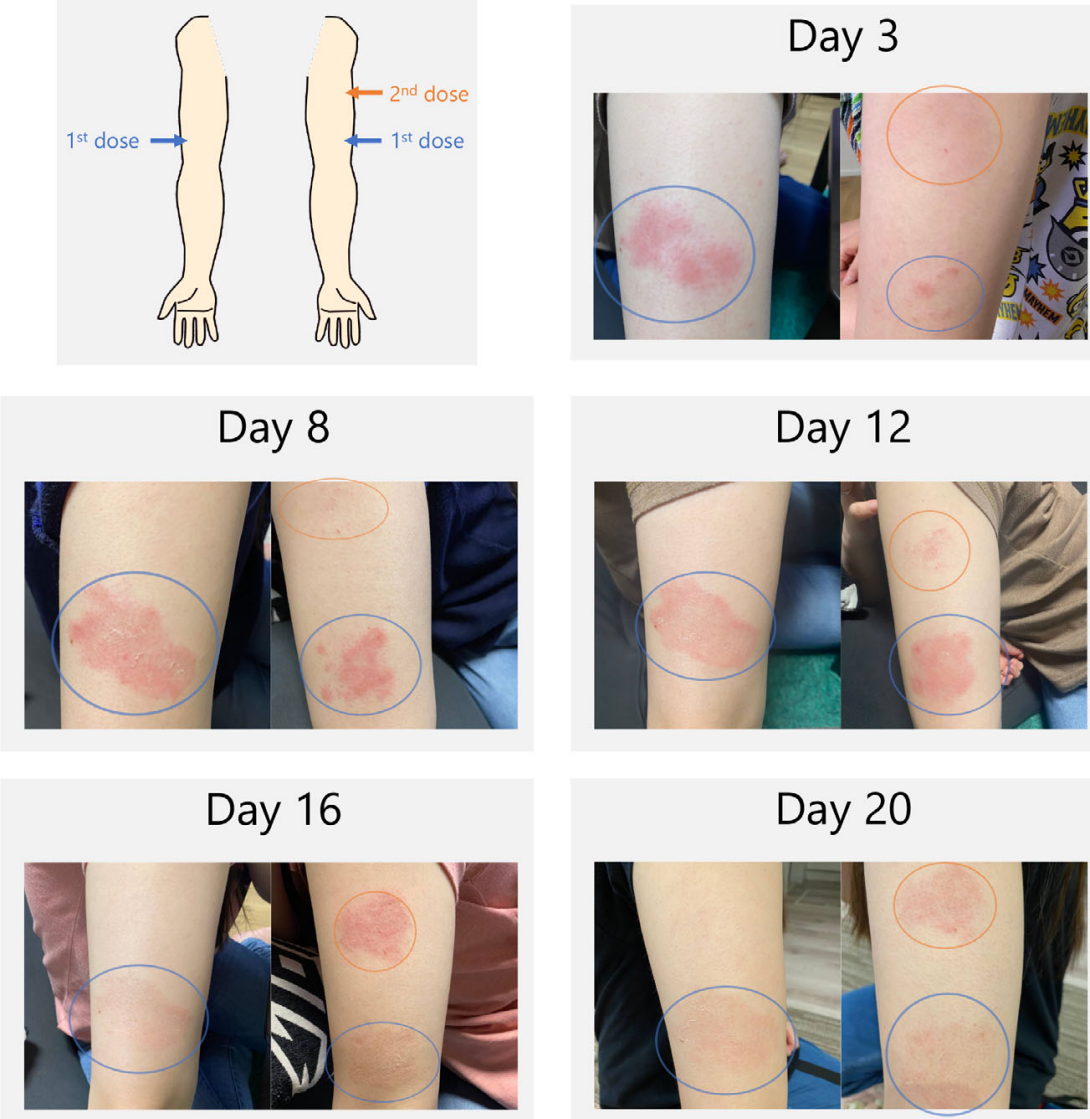
Delayed cutaneous reaction to dupilumab. The first dose of dupilumab was administered distally to the upper regions of both arms (site indicated by blue circles). The second dose of dupilumab was administered in the upper proximal region of the left arm (site of administration is indicated by the orange circle). The skin rash at the site of the first dose (blue circle) appeared on day 3 and disappeared after peaking on day 8. The skin rash at the site of the second dose (orange circle) appeared on the 12th day and disappeared after peaking on the 16th day

A 30‐year‐old woman presented with severe asthma, poorly controlled despite treatment with fluticasone furoate 200 μg, umeclidinium bromide 62.5 μg, vilanterol 25 μg and frequent prednisolone burst administration; we initiated treatment with dupilumab. The respiratory symptoms improved dramatically with no adverse events. Three days after the second dose of dupilumab, a skin rash appeared at the site of the first dose. The rash gradually worsened; however, it resolved spontaneously without treatment. Twelve days after the second dose, a similar rash was observed at the site of the second dose, which also resolved spontaneously (Figure 1). Dupilumab contains polysorbate 80 (PS80) as a base agent. Polyethylene glycol, cross‐reactive with PS80, is also used as a stabilizer of mRNA in coronavirus vaccines.^1^ There are reports of delayed injection site reactions similar to the present case^2^; however, these occurred at the site of the most recent injection. In this case, the skin rash appeared at the site of the first injection 3 days after the second injection, rather than at the site of current administration. The rash may be a reaction to subcutaneous residual material of polysorbate metabolites.

## CONFLICT OF INTEREST

None declared.

## AUTHOR CONTRIBUTION

Toshiyuki Sumi: conception and design. Toshiyuki Sumi, Yuta Nagahisa, Keigo Matsuura, Motoki Sekikawa and Yuichi Yamada: acquisition of clinical, radiological and pathological data, and drafting the article. Hisashi Nakata and Hirofumi Chiba: revision of intellectual content and final approval of the version to be published. All authors read and approved the final manuscript.

## ETHICS STATEMENT

The authors declare that appropriate written informed consent was obtained for publication of this case report and accompanying images.
